# Related factors for depression among Chinese men who have sex with men: A protocol for systematic review and meta-analysis

**DOI:** 10.1097/MD.0000000000024516

**Published:** 2021-02-19

**Authors:** Yuping Zheng, Jing Gao, Xiaolin Jiang

**Affiliations:** Chengdu University of Traditional Chinese Medicine, Chengdu, Sichuan Province, China.

**Keywords:** China, men who have sex with men, meta-analysis, related factors

## Abstract

**Background:**

Men who have sex with men (MSM) are the focus group of acquired immune deficiency syndrome intervention, because of the impact of social concepts, they suffer from a lot of social discrimination, and mental health problems are more prominent. Depression is prevalent in Chinese MSM. The aim of this systematic review is to systematically assess the current evidence on factors associated with depression among Chinese MSM.

**Methods:**

This study will be conducted in strict accordance with the preferred reporting items for systematic reviews and meta-analysis protocols. China National Knowledge Infrastructure, VIP Database, Wanfang Database, Chinese Biomedical Literature Database, PubMed, Embase, Web of Science, and the Cochrane Library will be searched comprehensively to collect cross-sectional studies, case-control studies, Cohort studies for depression of the related factors among Chinese MSM. The retrieval time is limited from the establishment of the database to December 2020. Two researchers independently evaluate the study and extract the data according to the inclusion and exclusion criteria. Meanwhile, the Newcastle–Ottawa scale and the bias risk assessment criteria of the agency for healthcare research and quality will be used to evaluate the risk of bias in the included studies, and the meta-analysis will be carried out by using Stata 15.0.

**Results:**

This study will systematically evaluate the related factors for depression among Chinese MSM based on published studies. The results will be presented here.

**Conclusion:**

The conclusion of the systematic review and meta-analysis will provide clinical evidence for the related factors and prevention and treatment measures of depression among Chinese MSM.

## 1 Introduction

Men who have sex with men (MSM) refers to men who have oral or anal sex with the same sex, also known as male homosexual sex and male male contact.^[1]^ MSM has high-risk behaviors such as unprotected anal sex, multiple sexual partners, and commercial sex behaviors which increases the risk of human immunodeficiency virus (HIV) infection.^[2,3]^ Furthermore, many Chinese MSM also have unprotected sex with women, which increases the chance of contracting HIV to some extent.^[4]^ In our country, sexual transmission is the main way of acquired immune deficiency syndrome (AIDS) transmission.^[5]^ According to the data released by the Chinese AIDS prevention and control center in 2018, among newly infected HIV patients or AIDS patients, there are 9379 cases of homosexual transmission, accounting for 22.7%.^[6]^ Therefore, Chinese MSM have been listed as the focus of AIDS intervention population.^[7]^

MSM suffer a lot of social stigma and discrimination due to their sexual orientation and HIV infection, and their mental health problems are very serious.^[8]^ According to the study, Chinese MSM are under great psychological pressure to conceal their sexual orientation and to marry the opposite sex in order to cope with the responsibility of carrying on the family line influenced by traditional value.^[9,10]^ MSM has prominent mental health problems, especially depression.^[11]^ Meta-analysis shows that the incidence of MSM depressive symptoms is as high as 43.2%.^[12]^ And MSM with HIV are more likely to be depressed than those without the disease.^[13]^ Depressive symptoms can reduce adherence to medication in MSM with HIV and increase the risk of suicide.^[14,15]^ At present, a number of epidemiological investigations have been conducted on the related factors for depression among MSM, but the conclusions are not consistent due to the influence of sample size, region, and other confounding factors.

Therefore, this study will use the method of meta-analysis to systematically evaluate the related factors for depression among Chinese MSM, in order to provide medical personnel with higher quality clinical basis and put forward the reference measures to intervene the mental health problems of Chinese MSM.

## 2 Objectives

The purpose of this systematic review and meta-analysis is to solve the following problems:

(1) What factors may affect depression among Chinese MSM?
(2) What corresponding measures are proposed for the related factors for depression among MSM in China?

## 3 Methods

### 3.1 Protocol registration

This protocol has been registered on the INPLASY platform of systematic review and meta-analysis (https://inplasy.com/) with the registration number INPLASY2020110142 and DOI number 10.37766/inplasy2020.11.0142. This study will strictly follow the preferred reporting items for systematic review and meta-analysis protocols.^[16]^

### 3.2 Eligibility criteria

#### 3.2.1 Inclusion criteria

(1) Types of studies: cross-sectional, case-control, and cohort studies.
(2) Types of participants: MSM in China.
(3) Exposure factors: exposure factors are associated with depression among MSM, and the definition of each factor is the same or similar.
(4) Types of outcomes: we will select self-rating depression scale^[17]^ and center for epidemiologic studies depression scale^[18]^ as outcome indicators for this study. And specific outcome indicator are shown as odds ratios (OR) values and 95% confidence intervals (CI) of related factors.

#### 3.2.2 Exclusion criteria

(1) Duplicate publications and studies from the same data sources will be excluded.
(2) Studies that fail to obtain outcome indicators due to data missing and errors will be excluded;
(3) Reviews, commentaries, letters, case reports will be excluded.

### 3.3 Search methods

#### 3.3.1 Information sources

To find the studies of related factors for depression among Chinese MSM, 8 databases included China National Knowledge Infrastructure, VIP Database, Wanfang Database, Chinese Biomedical Literature Database, PubMed, Embase, Web of Science, and the Cochrane Library will be searched from the construction of the database to December 2020.

#### 3.3.2 Search strategy

According to the types of different databases, this study adopts the search strategies by combining subject words with free words, and Boolean logic operators, truncation operators, and so on are used for study search. Meanwhile, we traces the references included in the study and supplements the relevant research as far as possible. And the search keywords include “men who have sex with men OR MSM OR gay OR homosexuality OR faggotry,” “depression OR depressive symptom OR depressive disorder OR emotional depression,” “risk factors OR related factors OR influence factors OR root cause analysis OR relative factors analysis OR relevant research OR predictive factor,” “China OR Chinese,” et al. Taking PubMed as an example, the specific retrieval strategy is shown in Table 1.

**Table 1:** Search strategy of PubMed.

Number	Search terms
#1	Men who have sex with men
#2	MSM
#3	Gay
#4	Homosexuality
#5	Faggotry
#6	#1 OR #2 OR #3 OR #4 OR #5
#7	Depression
#8	Depressive symptom
#9	Depressive disorder
#10	Emotional depression
#11	#7 OR #8 OR #9 OR #10
#12	Risk factors
#13	Related factors
#14	Influence factors
#15	Root cause analysis
#16	Relative factors analysis
#17	relevant research
#18	Predictive factor
#19	#12 OR #13 OR #14 OR #15 OR #16 OR #17 OR #18
#20	China
#21	Chinese
#22	#20 OR #21
#23	#6 AND #11 AND #19 AND #22

### 3.4 Data management

With the help of the established search strategies, all the study data searched are imported into Endnote X9, a literature management software, which is convenient for researchers to filter and eliminate duplicate studies. For studies retrieved manually, such as the references tracked from included studies, will be imported into an Excel spreadsheet together with the final studies from Endnote X9 for further reviewing.

### 3.5 Selection process and data extraction

**Figure 1:**
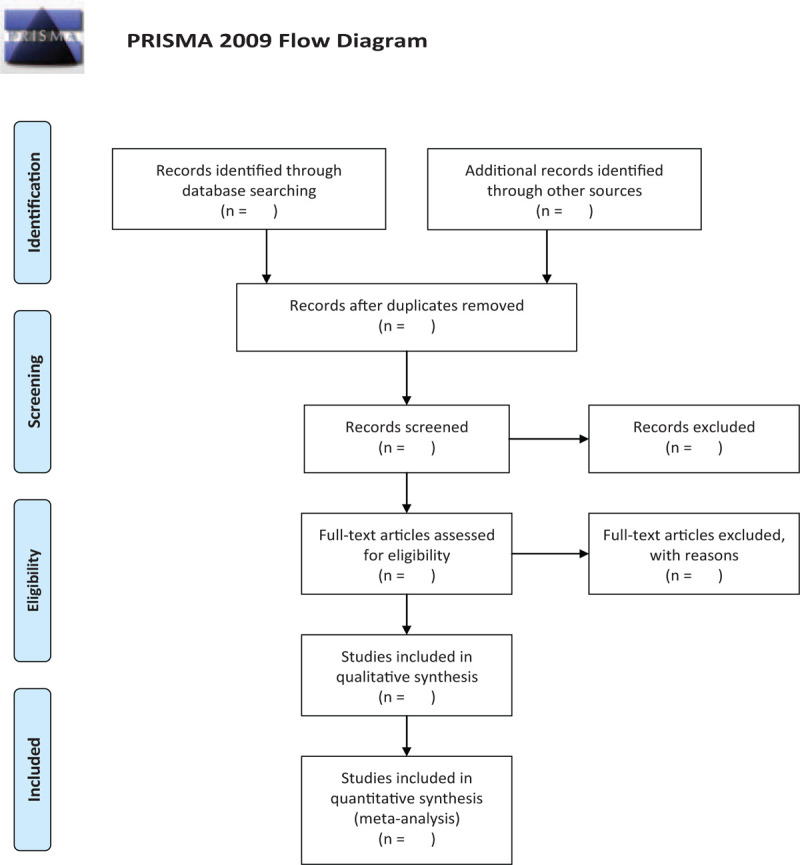
Flow chart of search process.

Two researchers independently select studies and collect data in strict accordance with inclusion and exclusion criteria. During the assessing process, any disagreement can be resolved through discussion between the 2 researchers or through arbitration by a third researcher. Studies screening includes 2 steps. First of all, screen the titles and abstracts to eliminate studies that are duplicate and that do not meet the inclusion criteria. When the title and abstract are not clear, then read the full text and finally determine the included studies. For study where full text is not available, we will try to contact the original author as much as possible to fill in the missing information. The flow chart (Fig. 1) will be applied throughout the selection process to document the reasons for the exclusion of each study and to show the number of studies that are eventually included.

Two researchers will independently collect data from the included studies, and the data will eventually be reviewed by a third researcher. The detailed information extracted in this study include:

(1) basic information of included studies: title, the first author, year of publication, study types, study area;
(2) baseline characteristics and exposure factors of the study participants: average age or age range, sample size, measuring tool, related factors for depression among MSM;
(3) the key elements of quality evaluation;
(4) the outcome indicators and data results obtained from the studies.

### 3.6 Quality assessment

To ensure the high quality of the study, we will use assessment tools to assess the risk of bias in order to assess the strength of evidence in related factors meta-analysis. In addition, the assessment will be carried out in accordance with a predetermined unified process. Two researchers independently evaluate the quality of the studies, and in case of disagreement during the evaluation, the dispute will be resolved through discussion by 2 researchers or be submitted to a third research for resolution. Depending on the types of studies we included, we will use the following 2 tools to assess the risk of bias. One is the Newcastle–Ottawa scale^[19]^ will be used for the risk assessment of bias in case-control studies and cohort studies. The total score of the scale is 9 points, among which studies with 6 points or more are defined as high-quality studies. And the other is the bias risk assessment criteria of the agency for healthcare research and quality^[20]^ that is used for the risk assessment of bias in cross-sectional studies. Each item is judged as “yes” for 1 point, “No” or “Unclear” for 0 points. The total score of the scale is 11 points, in which 0 to 3 points are defined as low-quality studies, 4 to 7 points as medium-quality studies, and 8 to 11 points as high-quality studies.

### 3.7 Statistical analysis

The effect size of related factors for depression among Chinese MSM are calculated as OR and 95% CI. The meta-analysis will be performed by using Stata 15.0, in which at least 2 included studies have the same related factors for depression among Chinese MSM. The *Q* test and *I*^*2*^ index will be applied to determine the heterogeneity of studies. If *I*^*2*^ > 50% with *P* < .10, which indicates the heterogeneity in the study, the data will be analyzed using the random-effects model. Or else, the fixed effects model will be applied for analysis

### 3.8 Sensitivity analysis

Sensitivity analysis will be performed by removing the included studies one by one in order to assess the changes of the overall effects after removing a certain study.

### 3.9 Publication bias

Publication bias will be assessed by using funnel plots, Egger (regression) tests, and the Begg test.^[21]^ When the results show the existence of publication bias, we will further evaluate the impact of bias on the results by using the trim and fill method.^[22]^

## 4 Discussion

Depression is a mental disease characterized by depressed mood and low mood and is caused by physiological, psychological, and social factors,^[23]^ and the incidence of suicidal ideation is high in severe cases.^[24]^ There is a close relationship between high-risk sexual behavior and psychosocial status of MSM.^[25]^ These factors are closely related to the occurrence of depression among Chinese MSM.

In this study, we will gather and synthesize the information on factors related to depression among Chinese MSM in the existing studies, and analyze it with the strict method of systematic review and meta-analysis, so as to identify the relevant factors that lead to depression among Chinese MSM and provide reliable scientific basis for the formulation of effective prevention and treatment measures.

## Acknowledgment

The authors are grateful to the Chengdu Science and Technology Bureau (Benefit the People Project) for funding this study.

## Author contributions

**Conceptualization:** Yuping Zheng, Jing Gao.

**Data curation:** Yuping Zheng, Jing Gao, Xiaolin Jiang.

**Formal analysis:** Yuping Zheng, Xiaolin Jiang.

**Methodology:** Yuping Zheng.

**Project administration:** Yuping Zheng.

**Writing – original draft:** Yuping Zheng.

**Writing – review & editing:** Jing Gao and Yuping Zheng.
